# Patient Preferences for Diabetes Treatment Among People With Type 2 Diabetes Mellitus in China: A Discrete Choice Experiment

**DOI:** 10.3389/fpubh.2021.782964

**Published:** 2022-02-01

**Authors:** Yuankai Huang, Qixiang Huang, Ailin Xu, Mengqing Lu, Xiaoyu Xi

**Affiliations:** The Research Center of National Drug Policy and Ecosystem, China Pharmaceutical University, Nanjing, China

**Keywords:** diabetes, discrete choice experiment, preferences, willingness to pay, Chinese

## Abstract

**Introduction:**

Preferences for diabetes treatment-related attributes may be significant in diabetes management. However, there is a lack of evidence on patient preferences for diabetes in China.

**Methods:**

A large-scale questionnaire survey was conducted in the hospitals of mainland China. Participants' preferences for six attributes were evaluated *via* a discrete choice experiment (DCE) using the conditional logit model. Patients' willingness to pay (WTP) for each attribute was calculated based on the cost attribute.

**Results:**

The sample consisted of 709 patients (male 51.9%; female 48.1%). The results of the model indicated that patients' preference weight (PW) of days on which the blood glucose level is under control per week was the highest (1.41), and the PW of blood glucose monitoring frequency was the lowest (0.642). Patients were generally willing to pay for improvements in their type 2 diabetes mellitus (T2DM) treatment, and they had relatively higher WTP to avoid the blood glucose level within a normal value of 1 day/week (¥176.01) and also to avoid the frequency of hypoglycemic events within the range of 1–2/month (¥144.53).

**Conclusion:**

The number of days on which the blood glucose level is under control per week is the most important attribute in the treatment choice for patients with T2DM in China, followed by the frequency of hypoglycemic events, medication regimen, weight change, and blood glucose monitoring.

## Introduction

Diabetes has become a major health problem worldwide due to multiple reasons, including the rapid growth of the world economy, changes in people's lifestyles, and the aging of the population. Globally, in 2017, there are 425 million adults (age 20–79 years) with diabetes, with an estimated prevalence of 8.8% (1). The situation of China is also severe. The number of adult patients with diabetes in China is as high as 114 million, which ranks the first in the world and will expectedly increase to 120 million by 2045 (1). In China, the prevalence of diabetes has increased from 2.51 in 1994 to 10.9% in 2013, while the prediabetes prevalence rate has increased from 3.20 to 35.7% (2, 3). Diabetes has become the eighth leading cause of death in 2017 in China (4). Diabetes and its associated chronic complications have become one of the most important health burdens for the Chinese public (5).

In China, type 2 diabetes mellitus (T2DM) is the dominant type of diabetes, which accounts for more than 90.0% of the overall diabetic population (6). In the past few decades, the effectiveness and safety of therapies for diabetes have been tremendously improved in China, and patient adherence to treatment may have become a major factor affecting the quality of long-term blood glucose control. Existing studies have shown that patient preferences toward factors other than the effectiveness of their diabetes treatment can also affect their compliance to a certain extent, which in turn can alter the treatment effectiveness (7). Figuring out what patients expect and value for the regiments of managing chronic diseases, like diabetes, is increasingly significant in the design and delivery of diabetes treatment (8). Therefore, the healthcare providers need to recognize and well-utilize the characteristics of the treatment that patients want to incorporate into their diabetes management (9). The optimal diabetes treatment for each individual patient should be both clinically effective and consistent with the patient's needs and preferences.

Existing studies focus on patient preferences for the treatment of diabetes in developed countries, and several studies have shown that a variation of socioeconomic and cultural backgrounds among populations may lead to differences in patient preferences (10, 11). However, there is a lack of evidence on patient preferences for diabetes in developing countries with low levels of economic development, and specifically, there is no such study in China, a developing country with a large diabetic patient population.

Based on the above-mentioned reasons, this study aims to assess the preferences for diabetes treatment among patients with T2DM in China, to deepen the understanding of the health preferences of Chinese patients with diabetes, and to help medical staff implementing the targeted disease management scheme. To improve the applicability of the results, this study introduced discrete choice experiments (DCEs) to measure the preferences for each of the important attributes of the treatment by asking respondents to choose one set of attributes from a number of presented sets of attributes that simulate real situations (12). When cost is included as an attribute in the DCE, the results can be used to calculate the willingness to pay (WTP) for the attributes being measured (13)_._ The results of this study would allow health professionals to quantitatively compare patients' preference for specific treatments in China based on the evaluation of the attributes of each treatment.

## Methods

### Discrete Choice Experiment

A discrete choice experiment draws upon Lancaster's economic theory of value (14) and the random utility theory (15, 16). This technique is used in market research to assess how consumers value the underlying attributes that comprise different kinds of products and make choice decisions. The last few decades have seen an increasing use of this technique in health economics (17, 18). The decision-making process within a DCE is seen as involving a comparison of indirect utility functions (19). In making a series of choices, in each case, the subject chooses the option that leads to a higher level of utility.

### Sampling

In this study, the inclusion criteria were: (1) patients have a self-reported physician's diagnosis of T2DM for 2 years or longer; (2) patients used antidiabetic medications in the past 2 weeks; (3) patients aged 18 years or older; and (4) patients be able to and willing to participate in this study, and willing to sign the informed consent.

The minimum sample size was calculated using the formula for estimating the sample size requirement for DCE (20):


(1) $$\begin{array}{l} \text{n}=500c/\left(\text{t}\times \;\text{a}\right). \end{array}$$

In this formula, “*c*” is the largest number of levels for any of the attributes, “*t*” is the number of choice sets, and “*a*” is the number of alternatives in each choice set. And, for this study, the minimum sample size was 500 × 4/(8 × 2)=125.

A stratified sampling strategy was used in this study: (1) all 31 provincial administrative regions (including provinces, autonomous regions, and municipalities directly under the central government) in mainland China were covered in the sampling; (2) cities in each province/autonomous region or districts in each municipality were evenly divided into three groups according to their 2017 per capita gross domestic product, thereby generating 93 groups; (3) within each group, one city or district was selected using the random number method, thus 93 cities or districts were selected; (4) in each selected city or district, 1–2 secondary hospitals and 1–2 tertiary hospitals (primary healthcare facilities were excluded because, under the current hierarchical healthcare system of China, only patients with light symptoms or health problems are encouraged to visit primary facilities, but all the patients are accessible in secondary and tertiary facilities) were surveyed based on the hospital administrators' permission to conduct the survey, and the hospital level was verified by consulting the hospital information tool by National Health Commission of China (http://61.49.18.120:9090/unit/index). This ensured that 186–372 hospitals would be selected. In each surveyed hospital, two participants who met the inclusion criteria mentioned above were surveyed. Overall, 744 questionnaires were distributed.

### Survey Design

In accordance with the International Society for Pharmacoeconomics and Outcomes Research (ISPOR) good practices for a conjoint analysis in health, the survey instrument in this study was developed using the following steps: (1) the identification of medication attributes and levels; (2) medication attribute and level selection; and (3) experimental design (21). The questionnaire used in this survey consisted of the following parts: (1) the introduction of this survey; (2) questions of the patient's current conditions about the disease, like the type of disease, course of the disease, and disease complications; (3) a preference survey section; and (4) other sociodemographic information questions, like gender, age, education level, monthly income, and exercise frequency.

For the identification of attributes and levels, a comprehensive literature review was performed with an aim to identify, select, and summarize existing research until March 2018 that assessed patient preferences for T2DM for the attributes of antidiabetic treatments and/or their WTP using DCE. Keywords (“Diabetes Mellitus,” “Type 2 Diabetes Mellitus,” “Disease management,” “Willingness to Pay,” “DCE,” “Discrete Choice Experiment,” “Patient Preference,” “Conjoint,” and “Conjoint Analysis”) in English and Chinese were used. The search was carried out in a pool of databases (Medline, Web of Science, SpringerLink, Elsevier ScienceDirect, Wiley Online Library, CNKI, CSPD, and VIP).

After excluding the duplicate papers, 34 studies were collected. Then, by manually excluding the studies that were not in accordance with the description of the abovementioned literature, 15 studies were collected. (22–36) Among the studies using the DCE, the most frequently studied attributes were as follows: the control of glycated hemoglobin (HbA_1c_) level/days on which the blood glucose level is under control per week (*n* = 14; 93.33%), (22–35) the frequency of hypoglycemic events (*n* = 14; 93.33%), (22–35) weight change (*n* = 13; 86.67%), (22–24, 26–34, 36) medication regimen (*n* = 10; 66.67%), (22, 23, 25–27, 31–33, 35, 36) cost (*n* = 9; 60.00%), (22–24, 26, 27, 29, 31, 33, 35) blood pressure/heart function (*n* = 8;53.33%), (22, 23, 27, 28, 30, 31, 33, 34) and blood glucose monitoring (*n* = 4; 26.67%). (22, 23, 30, 31) An online consultation with a group of six clinical physicians who devoted more than 10 years to diabetes treatment was made to select the attributes and levels throughout a group discussion. The result of such consultations was reached until all clinical physicians' consensus on the selection or exclusion of each attribute and each level. Totally, six attributes and 24 levels were identified.

Six attributes were eventually selected, and each of them had four levels. Theoretically, there were 4^6^ = 4,096 possible scenarios. An orthogonal experimental design was carried out to generate the DCE design that was in compliance with the desirable design properties, like orthogonality, level balance, utility balance, and minimum overlap (37). After the design, 16 choice sets containing 32 scenarios were produced. Each respondent would answer these choice set questions (shown in Supplementary Materials), and their answers were used to analyze the preference and WTP for each attribute and level.

During April and May 2018, a pretest of the questionnaire was conducted in Nanjing, Changzhou, and Yangzhou, and 43 valid questionnaires were collected. The results showed that the questionnaire had acceptable understandability and readability.

Table 1 presents the abbreviated names, definitions, and levels selected in this study.

**Table 1:** Attributes and levels for the questionnaire.

**Name**	**Definition**	**Levels**
**Convenience attributes**		
Medication regimen	Mode and frequency of administration	Injection two times a day in relation to meals Injection once a day in relation to meals Injection once a day irrespective of meals Oral antidiabetics (OAD) up to three times a day without injection
Blood glucose monitoring frequency	Frequency of blood glucose monitoring	Once a day Three times per week Once a week No need for monitoring test
**Clinical outcome attributes**		
Weight change	Weight change in the first 6 months	Gain 3 kg/6 months Remain the same/6 months Lose 3 kg/6 months Lose 6 kg/6 months
Hypoglycemic event frequency	Frequency of occurring hypoglycemic events	Usually occur (1–2/month) Sometimes occur (1–2/three months) Occasionally occur (1–2/six months) Nearly not occur
Blood glucose controlled days	Number of days per week glucose level will be within normal range	1 day/week 3 days/week 5 days/week 7 days/week
**Other attributes**		
Cost	Payment per month	¥ 100 ¥ 70 ¥ 40 ¥ 0

### Data Collection

About 279 undergraduate students majoring in pharmacy-related disciplines were recruited as data collectors. Then, they were trained with the backgrounds, purposes, and goals of this study, as well as the procedures, the etiquette, and the techniques of performing the survey. Every three of the data collectors worked as a group for the data collection in one sampled city or district.

One group of data collectors randomly visited one of the secondary or tertiary hospitals in this city or district, and if the verbal permission of the dean/deputy director of this hospital was obtained, the data collector would conduct the survey, and if the verbal permission was not obtained, they would randomly visit another hospital, until the expected number of hospitals was surveyed. To conduct the survey, the data collectors randomly accessed a patient and orally introduced the background, content, purpose, and inclusion criteria of the survey to patients with diabetes who were leaving the endocrinology department. Patients who met the inclusion criteria and signed the informed consent were asked to complete a self-administered questionnaire. If the accessed patient did not meet the inclusion criteria and refused to participate or sign the informed consent, the data collector would randomly access another potential participant until the expected number of participants was surveyed.

Using an online survey system on mobile phones or tablet computers, the data collectors orally interviewed participants with each item of the questionnaire and recorded their responses, and then the survey system converted the data into electronic documents. The data collectors were not only allowed to provide any view on the questionnaire, but also the requirements or instructions of questionnaire filling. The survey system allowed the users to set restrictions on the format of responses and ensured the quality of data. About 15 postgraduates were recruited and trained to review the uploaded documents and immediately return those with data entry errors or data damages, which were corrected through return visits by data collectors whenever possible. The survey was carried out between July and August 2018.

### Statistical Analysis

The conditional logit model was used to analyze the results of choice sets. Each choice set had two alternatives, and the alternatives were assigned 0 if the respondent did not choose this alternative and 1 if he or she chose the alternative in the choice set. Within the model calculation, an effects coding system [instead of a dummy coding, the reference categories were estimated as the negative sum of the included categories (38, 39)] was used for five attributes other than cost attribute, which was defined as a numeric continuous variable based on the presence of a linear relationship between the levels for it. Similar to some other studies using the DCE, (22, 23) this study divided the attributes into two groups for analysis because the attributes related to the type of treatment are theoretically correlated with the attributes related to the outcomes of the treatment and this may lead to an inaccuracy in the estimation. The two groups are: (1) medication regimen, blood glucose monitoring frequency, and cost and (2) weight change, hypoglycemic event frequency, blood glucose controlled days, and cost. The cost was included in both groups for estimating the WTP for all attributes.

Coefficients estimated from the model can be interpreted as the relative strength of preference for each attribute level (21, 23). The difference between the coefficients for the best and worst levels of an attribute could be interpreted as the relative preference weight (PW) for that attribute. (24, 28) The relative importance (RI) of an attribute was obtained from the quotient between its PW and the sum of PWs of the five attributes other than cost (34). Based on the economic theory of demand, Willingness to pay for the attribute levels was calculated using the estimated coefficients divided by the coefficient of cost. (22, 26, 31) For the first level in each attribute, WTP should be interpreted as the “maximum amount of money a patient was willing to pay to experience this level.” For other levels, if WTP was positive, WTP should be interpreted as “maximum additional amount of money a patient was willing to pay to experience this level instead of the first level,” and if WTP is negative, its absolute value is presented in (**Table 4**) and should be interpreted as “maximum additional amount of money a patient was willing to pay to avoid experiencing this level instead of the first level.” Just to be clear, in the cases that x was negative, the descriptions of the levels were reversed (avoid …) to make them understandable.

Stata/MP statistical software version 13.0 was used to conduct all statistical analyses.

### Ethics Approval and Funding

All participants in this study signed the informed consent. The study protocol was reviewed and approved by the Ethics Committee of China Pharmaceutical University to ensure that the study was in accordance with the ethical guidelines of clinical investigation in China (as shown in Supplementary Materials). The study was funded by The Project of “Double First-Class” Construction of Discipline Innovation Team, China Pharmaceutical University (CPU2018GY39).

## Results

### Respondent Characteristics

A total of 709 patients with T2DM were surveyed. In Table 2, the demographics of the sample are summarized. The results showed that gender and most of the other characteristics of samples were evenly distributed.

**Table 2:** Respondent characteristics.

**Characteristic**	***n* (%)**
**Gender**	
Female	341 (48.1)
Male	368 (51.9)
**Age**	
18–30	6 (0.8)
31–40	32 (4.5)
41–50	162 (22.8)
51–60	184 (26.0)
61–70	198 (27.9)
71–80	110 (15.5)
>80	17 (2.4)
**Duration of diabetes**	
2–5 years	308 (43.4)
5–10 years	202 (28.5)
>10 years	199 (28.1)
**Frequency of blood glucose monitoring**	
Once a day	132 (18.6)
Three times per week	153 (21.6)
Once a week	238 (33.6)
Once a week	186 (26.2)
**Complication**	
None	420 (59.2)
Present	289 (40.8)
**Monthly income**	
< ¥1000	144 (20.3)
¥1000–2000	54 (7.6)
¥2000–3000	112 (15.8)
¥3000–4000	136 (19.2)
≥¥4000	263 (37.1)
**Frequency of exercise**	
Often	250 (35.3)
Sometimes	227 (32.0)
Occasionally	232 (32.7)

### Results of PWs for China

Analysis results from the model are provided in (Table 3). The chi-squared value of the model likelihood ratio test was statistically significant (*p* < 0.05). In addition, the estimated coefficients were all statistically significant (*p* < 0.05) except for the “remain the same/6 months” and “lose 3 kg/6 months,” indicating that patient preferences for these levels compared to the first level of their attributes were unclear. Cost was statistically significant (*p* < 0.05) in both models, indicating that cost significantly influenced patients' choice of treatment.

**Table 3:** Model analysis results.

**Attribute**	**Patients (*n* = 709)**		
	**Coefficient**	**SE**	**95% CI**	
**Medication regimen**				
Injection two times a day in relation to meals	−0.446	-	-	-
Injection once a day in relation to meals	0.166^***^	0.0319	0.103	0.228
Injection once a day irrespective of meals	−0.199^***^	0.0327	−0.263	−0.134
OAD up to three times a day without injection	0.479^***^	0.0332	0.414	0.544
**Blood glucose monitoring frequency**				
Once a day	−0.236	-	-	-
Three times per week	−0.276^***^	0.0327	−0.340	−0.211
Once a week	0.107^**^	0.0328	0.0424	0.171
No need for monitoring test	0.405^***^	0.0328	0.341	0.470
Cost	−0.00610^***^	0.000512	−0.00711	−0.00510
**Weight change**				
Gain 3 kg/6 months	−0.309	-	-	-
Remain the same/6 months	−0.0160	0.0347	−0.0841	0.0520
Lose 3 kg/6 months	−0.0486	0.0338	−0.115	0.0175
Lose 6 kg/6 months	0.374^***^	0.0354	0.304	0.443
**Hypoglycemic event frequency**				
Usually occur (1–2/month)	−0.753	-	-	-
Sometimes occur (1–2/3 months)	−0.103^**^	0.0332	−0.168	−0.0379
Occasionally occur (1–2/6 months)	0.429^***^	0.0352	0.360	0.498
Nearly not occur	0.427^***^	0.0347	0.359	0.495
**Blood glucose controlled days**				
1 day/week	−0.917	-	−	−
3 days/week	0.217^***^	0.0334	0.151	0.282
5 days/week	0.212^***^	0.0335	0.146	0.278
7 days/week	0.488^***^	0.0355	0.419	0.558
Cost	−0.00521^***^	0.000536	−0.00626	−0.00416

*SE, standard error; CI, confidence interval.*

^*^p < 0.05,

** *p < 0.01,*

*** *p < 0.001*.

The PW and RI of each attribute other than cost were calculated. The PW and corresponding RI values were the highest for three attributes: blood glucose controlled days (PW = 1.41; RI = 29.1%), the frequency of hypoglycemic events (PW = 1.18; RI = 24.4%), and medication regimen (PW = 0.926; RI = 19.1%), indicating that these attributes were relatively more concerned when patients with T2DM were choosing their treatment.

### Results of WTP

The calculated monthly WTP results are provided in (Table 4). The WTP analysis demonstrated that medication regimens without injection, the avoidance of blood glucose monitoring, the avoidance of hypoglycemic events, and the time at which the blood glucose level is under control were the more valued attributes of the treatment, and participants were willing to pay for these experiences or outcomes.

**Table 4:** Willingness to pay (WTP) for diabetes treatment.

**Unit change**	**Monthly WTP(¥)**
**Medication regimen**	
Avoid injection two times a day in relation to meals	73.11
Injection once a day in relation to meals	27.21
Avoid injection once a day irrespective of meals	32.62
OAD up to three times a day without injection	78.52
**Blood glucose monitoring frequency**	
Avoid one blood glucose monitoring test/day	38.69
Avoid three blood glucose monitoring tests/week	45.25
One blood glucose monitoring test/day	17.54
No need for monitoring test	66.39
**Weight change**	
Avoid gain 3 kg/6 months	59.39
Avoid remain the same	3.07
Avoid lose 3 kg/6 months	9.33
Lose 6 kg/6 months	71.79
**Hypoglycemic event frequency**	
Avoid usually occur (1–2/month)	144.53
Avoid sometimes occur (1–2/3 months)	19.77
Occasionally occur (1–2/6 months)	82.34
Nearly not occur	81.96
**Blood glucose controlled days**	
Avoid glucose level within normal range 1 day/week	176.01
Glucose level within normal range 3 days/week	41.65
Glucose level within normal range 5 days/week	40.69
Glucose level within normal range 7 days/week	93.67
Cost	Reference

## Discussion

This study aimed to examine patient preferences for the treatment of T2DM and their WTP for the treatment with different characteristics. Participants in our sample were patients with a self-reported physician's diagnosis of T2DM for more than 2 years. Their trade-offs for convenience of medication (medication regimen and blood glucose monitoring frequency), clinical outcomes of medication (weight change, hypoglycemic event frequency, and blood glucose controlled days), and the cost of medication were analyzed. As the results indicated, the most valued treatment attribute was blood glucose controlled days, followed by the frequency of hypoglycemic events, medication regimen, weight change, blood glucose monitoring, which means Chinese patients with T2DM believed that a steady, normal blood glucose level was more important than other treatment factors. Also, in this study, some interesting phenomena were identified.

Weight gain is a common symptom of T2DM, and as a well-known and sometimes severe side-effect of the treatment for diabetes, (40) a hypoglycemic event, such as weight loss, is a major concern for many patients, which may contribute to optimal treatment (41, 42). Mohamed et al. performed an analysis of preferences for oral antidiabetics (OAD) in Sweden and Germany and demonstrated that weight gain was the most important attribute, followed by glucose control (24). BØgelund et al. showed that, for Danish patients, weight change was the most important attribute (22). Similarly, Morillas et al. found that weight gain was the most important factor affecting patients' and physicians' medication choices in Spain and Portugal (23). In the UK, Gelhorn et al. proved that weight change was of primary importance to patients in their OAD preferences in T2DM. (34) One possible explanation for patients' concerns of weight change is that patients usually believe that weight gain will hamper blood glucose control and lead to an increasing risk in other diabetes-related complications in the long term, such as cardiovascular risk, and weight loss will lead to weakening and dysfunction of the body. However, the results of this study showed that the weight gain and a slight weight loss were not specifically concerned by patients with T2DM in China. A few studies showed that preferences were related to the cultural background of the study samples (43). Similar to our study's results, Brooks et al. in a recent preference study about two kinds of OAD in Japan found that according to patients' thought, the weight loss effect was the least important attribute (44). It is possible that, due to the regional and cultural differences in people's concepts and thoughts, (45) a mild or moderate weight change was of less importance to the East Asian population than to the European population.

Specific to the levels of each attribute, most of their estimated coefficients were in a monotone ascending or descending order as expected, but some of the levels did not comply with this order, such as comparisons of adjacent levels for the frequency of hypoglycemic events (occasionally vs. nearly not) and blood glucose controlled days (3 vs. 5 days/week). This phenomenon could be due to that patients would value the specific levels based on realistic factors rather than their hypothetical preference. Many possible realistic factors may influence patient preferences, such as whether the primary healthcare institutions in China were capable of providing management regiments with such levels of attributes and their quality, or whether the higher level of an attribute contained significantly added value compared to the lower level of the same attribute in the real world.

There was another unusual phenomenon in our study. For the attribute of medication regimen, the coefficients of injection once a day in relation to meals and injection once a day irrespective of meals were reversed totally (0.166 and −0.199, respectively). It was demonstrated that patients preferred injections in relation to meals and did not prefer injections irrespective of meals, and the possible reason was that patients' belief of injections in relation to meals could bring better blood glucose control.

The results of WTP calculation presented in this article generally demonstrated that patients were willing to pay for improved T2DM treatment, to gain additional benefits, like Jendle et al. stated in their study (31). Regarding the medication regimen, OAD was preferred to injections, and the WTP for OAD without injection was almost the same as WTP for avoiding injection two times a day in relation to meals, and three times as high as WTP for injection once a day in relation to meals. The results showed that patients preferred treatment with no need for the blood glucose monitoring test. Regarding a weight change in small range (a gain of 3 kg or a loss of 3 kg/6 months), Chinese patients were willing to avoid it. For a greater degree of weight loss (a loss of 6 kg/6 months), patients started to have WTP, which might be the influence of social and cultural backgrounds. The two highest WTP demonstrated that patients paid much attention to the frequency of hypoglycemic events and blood glucose control, and they were not desirable for frequent frequency of hypoglycemic events and the instability of blood glucose level.

As for the limitations of this study, it may have a bias in the sampling. The sampling procedure was not random because, when the data collectors were unable to survey in one of the randomly visited hospitals or survey one of the randomly visited potential participants, they would randomly access another. Additionally, only two participants were surveyed in each hospital because the research resources were limited, and covering the variance between the participants in different cities was usually important in surveys in China. Therefore, the final sample of this study may not represent Chinese patients with T2DM. Despite its wide use in health economics, DCE still has drawbacks. DCE is a technique for measuring the stated preferences and, even though it resembles the real situations as much as possible, it is still different from the decision process in real-world situations. Patients' choices for hypothetical treatment may not be the same as actual choices. The conditional logit model assumes that the measured utility is equal across all respondents and choice questions, (23) and therefore it does not consider variations in the preferences that arise from differences in individual characteristics, such as age, education, gender, and health status among respondents (preference heterogeneity) (21). In the DCE, the cognitive burden may increase with increases in the number of choice sets, (46) so that in our study respondents exposed to 16 choice sets may have a higher response variance that may influence the final results. In addition, the questionnaire just selected the attributes that were frequently used in previous studies and did not consider the other possible factors that might affect treatment preferences. Also, the attributes used in this study were generated based on the literature, and evidence from patients or residents were not considered because evidences collected in the design period of this study were of low quality and applicability, the suggested attributes were usually too general, too specific, or similar to the attributes from theliterature.

In this patient preference study, blood glucose controlled days are the most important attribute in the treatment choice for patients with T2DM in China, followed by the frequency of hypoglycemic events, medication regimen, weight change, and blood glucose monitoring. The results of this study can deepen the understanding of the preferences for patients with T2DM and to further assist in the development of diabetes treatment. Also, the results could be a boost of PRO use in clinical decisions for patients with T2DM in China, encouraging clinicians to take patient preferences for the treatment into consideration, which draws limited attention in China.

Preferences for different subgroups remain to be analyzed in future studies, and other attributes, such as heart function and gastrointestinal issues, may be taken into account as well.

## Data Availability Statement

The raw data supporting the conclusions of this article will be made available by the authors, without undue reservation.

## Ethics Statement

The studies involving human participants were reviewed and approved by Ethics Committee of China Pharmaceutical University. The patients/participants provided their written informed consent to participate in this study.

## Author Contributions

YH, QH, and XX contributed to study design, data analysis, interpretation, and manuscript drafting and review. AX and ML contributed to data collection and manuscript review. All authors approved the final version of the manuscript before its submission.

## Funding

The study was funded by The Project of “Double First-Class” Construction of Discipline Innovation Team, China Pharmaceutical University (CPU2018GY39).

## Conflict of Interest

The authors declare that the research was conducted in the absence of any commercial or financial relationships that could be construed as a potential conflict of interest.

## Publisher's Note

All claims expressed in this article are solely those of the authors and do not necessarily represent those of their affiliated organizations, or those of the publisher, the editors and the reviewers. Any product that may be evaluated in this article, or claim that may be made by its manufacturer, is not guaranteed or endorsed by the publisher.
