# Supplementary material for: Patient Preferences for Diabetes Treatment Among People With Type 2 Diabetes Mellitus in China: A Discrete Choice Experiment
Source: Front Public Health. 2022 Feb 1;9:782964. doi: 10.3389/fpubh.2021.782964 (PMC8846300; doi:10.3389/fpubh.2021.782964)
Supplement: Supplementary file 1 [file Data_Sheet_1.PDF]

Preference survey section in the questionnaire

Now, suppose there are two treatments for people with diabetes. If the effects of the treatments are same, which treatment would you prefer?

	**Treatment A**	**Treatment B**
**Mode of administration**	Injection once a day in relation to meals	Injection once a day irrespective of meals
**Blood glucose monitoring**	Three times per week	Once a week
**Cost**	¥ 0 per month	¥ 40 per month
**Which treatment would you prefer?**	○**Treatment A**	○**Treatment B**

	**Treatment A**	**Treatment B**
**Mode of administration**	Injection once a day in relation to meals	Injection once a day irrespective of meals
**Blood glucose monitoring**	Once a week	Three times per week
**Cost**	¥ 70 per month	¥ 100 per month
**Which treatment would you prefer?**	○**Treatment A**	○**Treatment B**

	**Treatment A**	**Treatment B**
**Mode of administration**	OAD up to three times a day without injection	Injection once a day in relation to meals
**Blood glucose monitoring**	Once a week	Once a day
**Cost**	¥ 0 per month	¥ 40 per month
**Which treatment would you prefer?**	○**Treatment A**	○**Treatment B**

	Treatment A	Treatment B
**Mode of administration**	Injection twice a day in relation to meals	OAD up to three times a day without injection
**Blood glucose monitoring**	No need for test	Once a day
**Cost**	¥ 70 per month	¥ 100 per month
**Which treatment would you prefer?**	○**Treatment A**	○**Treatment B**

	Treatment A	Treatment B
**Mode of administration**	Injection twice a day in relation to meals	OAD up to three times a day without injection
**Blood glucose monitoring**	Once a day	Three times per week
**Cost**	¥ 0 per month	¥ 70 per month
**Which treatment would you prefer?**	○**Treatment A**	○**Treatment B**

	Treatment A	Treatment B
**Mode of administration**	Injection once a day irrespective of meals	Injection twice a day in relation to meals
**Blood glucose monitoring**	No need for test	Three times per week
**Cost**	¥ 0 per month	¥ 40 per month
**Which treatment would you prefer?**	○**Treatment A**	○**Treatment B**

	Treatment A	Treatment B
**Mode of administration**	Injection twice a day in relation to meals	OAD up to three times a day without injection
**Blood glucose monitoring**	Once a week	No need for test
**Cost**	¥ 100 per month	¥ 40 per month
**Which treatment would you prefer?**	○**Treatment A**	○**Treatment B**

	Treatment A	Treatment B
**Mode of administration**	Injection once a day in relation to meals	Injection once a day irrespective of meals
**Blood glucose monitoring**	No need for test	Once a day
**Cost**	¥ 100 per month	¥ 70 per month
**Which treatment would you prefer?**	○**Treatment A**	○**Treatment B**

After treatment, the following different treatment effects are produced, which treatment would you prefer?

Treatment A	Treatment B
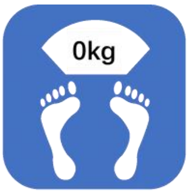	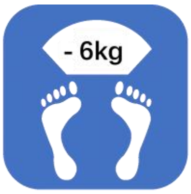
Remain the same/6 months	Weight lose 6 kg/6 months
Hypoglycemic events usually occur (1-2/month)	Hypoglycemic events occasionally occur (1-2/six months)
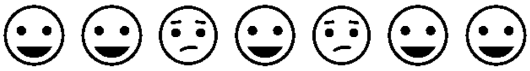	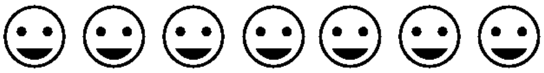
Blood glucose control for 5 days/week	Blood glucose control for 7 days/week
¥ 70 per month	¥ 40 per month

Which treatment would you prefer?

○**Treatment A** ○**Treatment B**

Treatment A	Treatment B
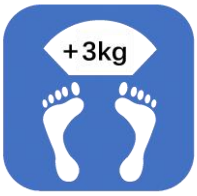	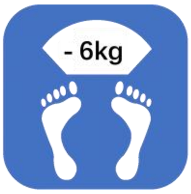
Weight gain 3 kg/6 months	Weight lose 6 kg/6 months
Hypoglycemic events usually occur (1-2/month)	Hypoglycemic events nearly not occur
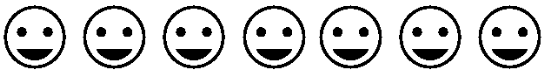	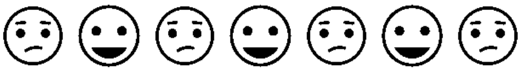
Blood glucose control for 7 days/week	Blood glucose control for 3 days/week
¥ 100 per month	¥ 70 per month

Which treatment would you prefer?

○**Treatment A** ○**Treatment B**

Treatment A	Treatment B
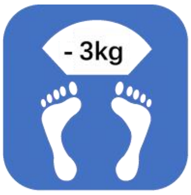	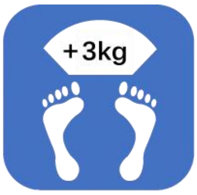
Weight lose 3 kg/6 months	Weight gain 3 kg/6 months
Hypoglycemic events nearly not occur	Hypoglycemic events sometimes occur (1-2/three months)
Blood glucose control for 1 day/week	Blood glucose control for 3 days/week
¥ 100 per month	¥ 0 per month
**Which treatment would you prefer?**	
○**Treatment A**	○**Treatment B**

Treatment A	Treatment B
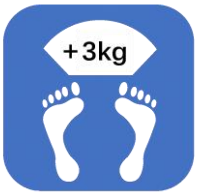	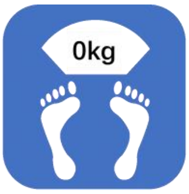
Weight gain 3 kg/6 months	Remain the same/6 months
Hypoglycemic events occasionally occur (1-2/six months)	Hypoglycemic events nearly not occur
Blood glucose control for 1 day/week	Blood glucose control for 7 days/week
¥ 70 per month	¥ 0 per month
**Which treatment would you prefer?**	
○**Treatment A**	○**Treatment B**

Treatment A	Treatment B
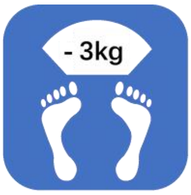<br><br>Weight lose 3 kg/6 months<br><br>Hypoglycemic events occasionally occur (1-2/six months)<br><br>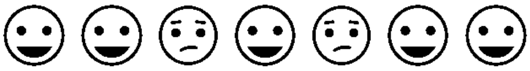<br>Blood glucose control for 5 days/week<br><br>¥ 0 per month	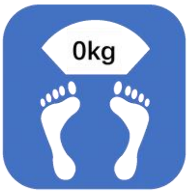<br><br>Remain the same/6 months<br><br>Hypoglycemic events sometimes occur (1-2/three months)<br><br>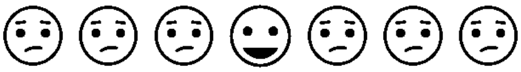<br>Blood glucose control for 1 day/week<br><br>¥ 40 per month
**Which treatment would you prefer?**	
○**Treatment A**	○**Treatment B**

Treatment A	Treatment B
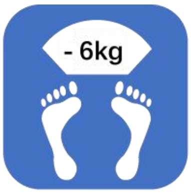<br><br>Weight lose 6 kg/6 months<br><br>Hypoglycemic events usually occur (1-2/month)<br><br>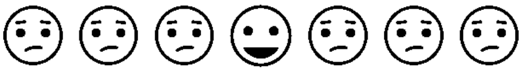<br>Blood glucose control for 1 day/week<br><br>¥ 0 per month	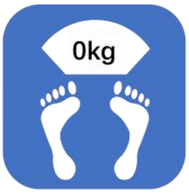<br><br>Remain the same/6 months<br><br>Hypoglycemic events occasionally occur (1-2/six months)<br><br>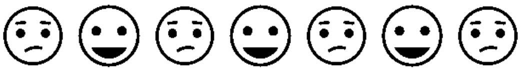<br>Blood glucose control for 3 days/week<br><br>¥ 100 per month
**Which treatment would you prefer?**	
○**Treatment A**	○**Treatment B**

Treatment A	Treatment B
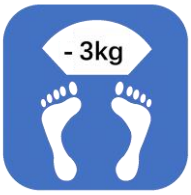 Weight lose 3 kg/6 months	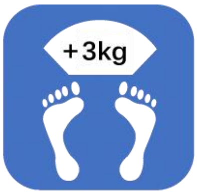 Weight gain 3 kg/6 months
Hypoglycemic events sometimes occur (1-2/three months)	Hypoglycemic events nearly not occur
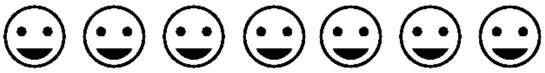<br>Blood glucose control for 7 days/week	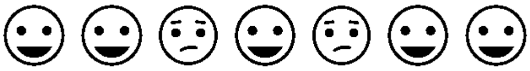<br>Blood glucose control for 5 days/week
¥ 70 per month	¥ 40 per month
**Which treatment would you prefer?**	
○**Treatment A**	○**Treatment B**

Treatment A	Treatment B
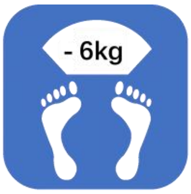 Weight lose 6 kg/6 months	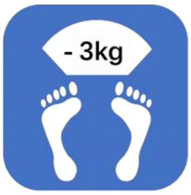 Weight lose 3 kg/6 months
Hypoglycemic events sometimes occur (1-2/three months)	Hypoglycemic events usually occur (1-2/month)
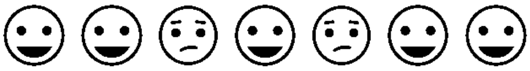<br>Blood glucose control for 5 days/week	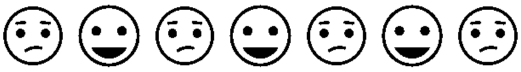<br>Blood glucose control for 3 days/week
¥ 100 per month	¥ 40 per month
**Which treatment would you prefer?**	
○**Treatment A**	○**Treatment B**
