# Supplementary material for: Patient Preferences for Diabetes Treatment Among People With Type 2 Diabetes Mellitus in China: A Discrete Choice Experiment
Source: Front Public Health. 2022 Feb 1;9:782964. doi: 10.3389/fpubh.2021.782964 (PMC8846300; doi:10.3389/fpubh.2021.782964)
Supplement: Supplementary file 2 [file Data_Sheet_2.PDF]

China Pharmaceutical University

IRB Approval

Serial Number: CPU2019015

Research Project	Clinical Pharmacy China by China Pharmaceutical University		
Research Categories	Questionnaire Inquiry	Applicant	National Licensed Pharmacist Development Research Center, China Pharmaceutical University
Sponsor	China Pharmaceutical University	Date	April 30, 2018
Principal Investigator	Xi Xiaoyu	Review Materials	As described in appendix
Review Channel	Review Conference ☐ Quick Review ■		
Contact Information of the Ethics Committee	Address: Science and Technology Department, China Pharmaceutical University, No.24 Tongjia Lane, Nanjing21198,China,Tel: 025-83271487		
Comments from Ethics Committee			

After being examined by this Ethics Committee, it is agreed to carry out the research.

Comments and Suggestions: None ■ Yes □

Will the research be subject to continuous review by Ethics Committee? Yes ■ No □

The review frequency is from the date of approval of the study: 3 months □ 6 months ■ 1 year □

The Ethics Committee has the power to change the frequency of continuous reviews according to actual progress.

Signature of Chairman: [illegible] Date: 2018. 4. 30

Note: (Please read carefully)

1. The approval document is valid for three years. Please continue to apply beyond the validity period.
2. This approval document will be filed with each central institution and its ethics committee. If the other party has different opinions on the feasibility of your institution (including the qualification and experience of researchers, equipment, conditions, etc.), please contact this Ethics Committee in time.
3. Approved projects shall be implemented in accordance with the plan approved by the Ethics Committee and in line with the principles of CFDA-GCP and the Helsinki Declaration.
4. Suspend/terminate the investigation in advance, please inform the Ethics Committee in time.
5. Please inform the Ethics Committee in time to re-examine any modification of the approved investigation plan, informed consent form and other materials and the replacement of major researchers, and implement them after obtaining approval.
6. If any violation of the experimental scheme is found, the Ethics Committee shall be informed in time.
7. According to the opinion of this Ethics Committee on the frequency of continuous review, whether the experiment starts or not, please apply for continuous review one month before the expiration of the continuous review date.
8. After completing the investigation, please submit the final report for review by this Ethics Committee.

Appendix

The list of approval materials for the Clinical Pharmacy China by China Pharmaceutical University is as follows:

No.	Documents	Version/Date
1	Application Form for Preliminary Examination	2018.4
2	Registration Form	2018.4
3	Resumes of Main Researchers and List of Participants	2018.4
4	Research Plan	2018.4
5	Informed Consent	2018.4
6	Investigators' Manual Documents	2018.4
7	Notes to Investigators' Manual Documents	2018.4
8	Questionnaire	2018.4
9	Respondents' Manual Documents	2018.4
10	Notice of Respondents	2018.4
11	Investigator Recruitment Materials	2018.4
11.1	Overview of Research and Progress Plan	2018.4
11.2	Investigator Qualification Certificate	2018.4
